# Analysis of risk factors of rapid attenuation of graft endothelium in the early stage after penetrating keratoplasty

**DOI:** 10.1371/journal.pone.0266072

**Published:** 2022-04-05

**Authors:** Ting-ting Xu, Rui Cao, Yan-ling Dong, Li-xin Xie, Jun Cheng

**Affiliations:** 1 Weifang Medical University, Institute of Clinical Medicine, Weifang, China; 2 State Key Laboratory Cultivation Base, Shandong Provincial Key Laboratory of Ophthalmology, Qingdao, China; 3 Eye Institute of Shandong First Medical University, Qingdao Eye Hospital of Shandong First Medical University, Qingdao, China; 4 School of Ophthalmology, Shandong First Medical University, Jinan, China; National Taiwan University Hospital, TAIWAN

## Abstract

This study aimed to analyze the factors of rapid attenuation of graft endothelium in the early stage after penetrating keratoplasty (PKP), with a view to guiding patients with PKP to better long-term outcomes. This study included 226 patients who underwent PKP with follow-up time >1 year at the Qingdao Eye Hospital of Shandong First Medical University from January 2018 to June 2020. Medical records were retrospectively studied, and donor factors, patient factors, and surgical factors were comparatively analyzed to clarify those affecting the rapid decay of graft endothelium after PKP. The median time between excision and death >60 min and patient age >60 years were risk factors for endothelial cell loss (ECL) rate >30% at 1 month postoperatively. However, a higher percentage of patients with donor age ≤60 years and Optisol preservation solution had endothelial cell density (ECD) >2000 cells/mm^2^ in the graft at postoperative 1 year. A year after the surgery, patients with corneal endothelial decompensation and immune rejection were at risk for ECD < 1000 cells/mm^2^. The combined operations had a significant effect on the ECL in the early postoperative period. Patients who underwent combined extracapsular cataract extraction or intraocular lens implantation had a significantly higher rate of ECL at postoperative 1 month than other patients, and no significant effect at postoperative 1 year. However, patients without combined operations have a higher probability of maintaining a high level of graft ECD. The graft diameter also affected postoperative ECL. In patients with a larger graft diameter, attenuation of ECD was slower. The ultimate goal of PKP is to maintain graft transparency for extended periods. The use of younger donors, minimizing unnecessary operation in the anterior chamber, and minimizing immune rejection may maintain a greater donor corneal endothelium in the long term.

## Introduction

A sufficient number and normal function of the corneal graft endothelium determine donor corneal transparency after penetrating keratoplasty (PKP) [1]. Chronic loss of the endothelium after PKP is one of the main reasons for graft failure [2]. All current efforts, such as improving corneal preservation solution, improving surgical technology, using stronger anti-rejection drugs, and strengthening patient follow-up, are committed to prolonging the survival time of corneal grafts. However, the exact mechanism of chronic loss of the graft endothelium remains unclear. Studies have shown that the fastest rate (up to 40%) of endothelial decay is 1 year post-surgery [3]. During the follow-up, we found that the attenuation of graft endothelium in some patients was very fast and had declined by more than 50% or even less than 1000 cells/mm^2^ within 1 year after surgery. In contrast, other patients can maintain a high endothelial cell density (ECD) for a long time. For example, Kayukawa et al. [4] reported that the graft ECD was more than 2000 cells/mm^2^ 5 years after PKP, indicating that the rate of graft decay determines the survival time of the graft after PKP. Therefore, this study analyzed the factors affecting the rapid attenuation of ECD after PKP to achieve better long-term outcomes for PKP.

## Materials and methods

This retrospective consecutive cohort study was approved by the Ethics Review Committee of the Qingdao Eye Hospital of Shandong First Medical University (Approval # Qingdao Eye Hospital Ethics [2021] No. 9). Verbal informed consent was obtained from each patient. The study protocol was conducted in accordance with the tenets of the Declaration of Helsinki.

The clinical data of patients who underwent PKP at the Qingdao Eye Hospital of Shandong First Medical University from January 2018 to June 2020 and followed up for more than 1 year were collected from the patients’ medical records. The exclusion criteria were incomplete clinical data and inadequate follow-up time.

Donor corneas were obtained from the Qingdao Red Cross Eye Bank, and the eye bank staff (CR) were responsible for collection and quality control. The harvesting and storage times were recorded. An eye bank specular microscope (Konan Eye Bank Kerato Analyzer, Model EKA-98; Konan Medical, Irvine, CA, USA) was used to examine the donor endothelium. Donor corneas with central ECD ≥ 2000 cells/mm^2^ were distributed for transplantation. Donor corneas were stored in D-X (prepared by Shandong Provincial Key Laboratory of Ophthalmology) or optisol-GS (Chiron Ophthalmics, Irvine, CA, USA) storage media at 4°C in our eye bank.

PKP was performed by four surgeons (XL, DY, ZH, and CJ). The donor graft ranged in size, from 0.25 to 0.5 mm larger than the host trephination. Other combined operations that may be performed under open sky include extracapsular cataract extraction (ECCE), pupilloplasty in ectopic pupils, synechiotomy in eyes with anterior or posterior synechiae, removal of the anterior chamber intraocular lens (IOL), and anterior vitrectomy in aphakic eyes. IOL implantation was performed after eight stitches of graft suturing.

During the first 3–5 days after surgery, intravenous hydrocortisone (2 mg/kg) was administered daily, after which oral prednisolone (1 mg/kg) was administered daily and tapered over a period of 2–3 months. Topical glucocorticoids were tapered from tobramycin dexamethasone to 0.1% and 0.02% fluminolone. Topical tacrolimus was administered three times daily to inhibit immune rejection. Patients with infectious keratitis continued to receive antifungal, antibacterial, and antiamoebic medications depending on the type of pathogen. Patients with fungal keratitis and acanthamoeba keratitis were forbidden to use glucocorticoids within 3 weeks after PKP, but were added according to the condition after 3 weeks.

Postoperative ECD was measured using a non-contact specular microscope at 1 week, 1 month, 3 months, 6 months, and 1 year after surgery. The endothelial cell loss (ECL) rate at 1 month postoperative = [(graft ECD before surgery–graft ECD at postoperative 1 month)/graft ECD before surgery] *100%.

### Statistical analysis

Descriptive statistical analysis was performed using SPSS version 26.0, and the measures in this study were normally distributed according to the Shapiro-Wilk test. Means between groups were expressed as mean ± SD by chi-square test, and one-way ANOVA was used to analyze the effect of ECD at postoperative 1 year. Multi-factor logistic regression analysis was used to analyze the risk factors of ECD attenuation, where *P* < 0.05 was considered a statistically significant difference.

## Results

### Demographic characteristics of patients

There were 226 cases comprising 149 men (65.9%) and 77 women (34.1%). The median age of the patients was 59 years (range, 15–88 years). The median follow-up time was 20 months (range, 12–35 months).

Infectious keratopathy (127 cases, 56.2%) was the main cause of PKP, of which fungal keratitis was the most common (61 cases, 27.0%), followed by herpes simplex keratitis (44 cases, 19.5%). Corneal endothelial decompensation was the leading cause of non-infectious keratopathy (47 cases, 20.8%), followed by keratoconus and corneal leukoplakia (both 17 cases, 7.5%) (Table 1). Additional procedures were performed in 41 (18.1%) eyes during PKP, mainly ECCE under open sky (28 eyes, 12.4%) and IOL implantation after graft suture (20 eyes, 8.8%). Immune rejection occurred in 15 (6.6%) patients within 1 year after surgery.

**Table 1:** Indications of penetrating keratoplasty.

Indications		n	%
**Infectious keratopathy**		127	56.2
	Fungal keratitis	61	27.0
	Herpes simplex keratitis	44	19.5
	Bacterial keratitis	19	8.4
	Amoebic keratitis	3	1.3
**Non infectious keratopathy**		99	43.8
	corneal endothelial decompensation	47	20.8
	Keratoconus	17	7.5
	Keratoleukoma	17	7.5
	Corneal stromal dystrophy	14	6.2
	Corneal graft asepticulcer	2	0.9
	Immune keratopathy	1	0.4
	Diabetic keratopathy	1	0.4

### Donor characteristics

The mean age of the donors was 57.3 ± 14.2 years (range, 18–90 years). A total of 72 and 154 grafts were preserved in the D-X fluid and in optisol, respectively. The median time between excision and death (DET) was 1.5 h (range, 0–16 h). The median time from death to transplantation (DTT) was 3 days (range, 0–9 days).

### ECD attenuation after PKP

The average rate of graft endothelial loss at 1 month after surgery was 21.1 ± 20.8% (range, -29.9% to 84.7%). In addition, 69 (30.5%) cases lost >30% of graft ECD at 1 month after the operation. There was a statistically significant difference between the mean ages of the ECD loss rate >30% and ≤30% groups (*P* = 0.044). The DTT in the ECD loss rate >30% group was significantly longer than that in the ≤30% group (*P* = 0.034). In contrast, patient age and DET were not significantly different between the two groups (Table 2).

**Table 2:** ECD Attenuation After PKP.

	ECL rate at 1 month postoperative		*P* Value	ECD at 1 year postoperative (cells/mm2)		*P* Value	ECD at 1 year postoperative (cells/mm2)		*P* Value
	>30% (n = 69)	≤30% (n = 157)		<1000 (n = 76)	≥1000 (n = 150)		≤2000 (n = 175)	>2000 (n = 51)	
**Patient age (years)**	60.33±1.62	56.63±0.91	0.07	59.53±1.28	55.17±1.20	0.024	57.47±0.98	53.75±2.22	0.087
**Donor age (years)**	60.20±1.41	57.34±0.95	0.044	58.57±1.48	56.77±1.21	0.405	58.56±1.06	53.14±1.97	0.016
**DET (min)**	151.87±25.45	132.08±11.85	0.269	125.61±19.88	135.37±14.77	0.698	138.15±13.32	111.27±25.86	0.344
**DTT (day)**	3.99±0.78	3.16±0.26	0.034	3.32±0.21	3.09±0.37	0.675	3.25±0.33	2.88±0.24	0.557

**Abbreviations:** PKP = Penetrating keratoplasty, ECD = Endothelial cell density, ECL = Endothelial cell loss,DET = time between excision and death, DTT = time from death to transplantation.

ECD < 1000/mm^2^ at postoperative 1 year was observed in 76 (33.6%) eyes, and patients in this group were significantly older (*P* = 0.024). Meanwhile, ECD > 2000/mm^2^ at postoperative 1 year was observed in 51 (22.6%) eyes, and the age of the donors in this group was significantly younger (*P* = 0.016) (Table 2). Corneal graft endothelial decompensation occurred in 22 (9.7%) eyes during the follow-up period, and the mean time of decompensation occurrence was 18.0 ± 8.4 months, including 15 patients with ECD <1000/mm^2^ at postoperative 1 year.

### Risk factors of rapid ECD attenuation after PKP

We analyzed the factors associated with ECL rate >30% at postoperative 1 month, ECD <1000/mm^2^ at postoperative 1 year, and ECD >2000/mm^2^ at postoperative 1 year into three aspects: donor, patient, and surgical factors.

#### Donor factors

Donor factors were analyzed in terms of donor age, DET, DTT, and preservation solution. DET > 60 min was a risk factor for an ECL rate of >30% at postoperative 1 month. The proportion of patients with high endothelial loss rates at postoperative 1 month and 1 year was greater at DTT > 3 days than at DTT ≤ 3 days, although the difference was not statistically significant. However, a higher percentage of patients with a donor age ≤60 years and an optisol preservation solution had ECD > 2000 cells/mm^2^ in the graft at postoperative 1 year (Table 3).

**Table 3:** Donor factors for graft endothelium attenuation.

		ECD loss rate of >30% at 1 month postoperative	Risk Ratio	*P* Value	ECD of <1000 cells/mm^2^ at 1 year postoperative	Risk Ratio	*P* Value	ECD of >2000 cells/mm^2^ at 1 year postoperative	Risk Ratio	*P* Value
**Age (years)**										
	>60	34/99 (34.3%)	1.246	0.492	34/99 (34.3%)	1.0384	0.355	18/99 (18.2%)	0.6998	0.016
	≤60	35/127 (27.6%)			42/127 (33.1%)			33/127 (26.0%)		
**DET (min)**										
	≤60	25/106 (23.6%)	0.643	0.044	36/106 (33.96%)	1.1018	0.697	33/106 (31.13%)	2.0753	0.349
	>60	44/120 (36.7%)			40/120(33.33%)			18/120(15.00%)		
**DTT (d)**										
	≤3	40/153 (26.1%)	0.6581	0.272	44/153 (28.8%)	0.656	0.248	37/153 (24.2%)	1.2607	0.636
	>3	29/73 (39.7%)			32/73 (43.8%)			14/73 (19.2%)		
**Preservation solution**										
	D-X	16/72 (22.2%)	0.6456	0.064	23/72 (31.9%)	0.9279	0.714	9/72 (12.5%)	0.4584	0.022
	Optisol	53/154 (34.4%)			53/154 (34.4%)			42/154 (27.27%)		

**Abbreviations:** ECD = Endothelial cell density, ECL = Endothelial cell loss, DET = time between excision and death, DTT = time from death to transplantation.

#### Patient factors

Patients aged > 60 years were a risk factor for ECL rate > 30% at 1 postoperative month and ECD <1000/mm^2^ at postoperative 1 year. A year after surgery, the proportion of patients with corneal endothelial decompensation with ECD < 1000 cells/mm^2^ was as high as 51.1%, which was significantly higher than that in other groups (29.1%, *P* = 0.004), while the percentage of ECD > 2000 cells/mm^2^ was only 8.5%, which was significantly lower in the other groups (26.3%, *P* = 0.01). Surprisingly, the rate of ECL was lower in patients with infectious keratopathy than in those with non-infectious keratopathy. Immune rejection within 1 year after surgery was also a risk factor for ECD < 1000 cells/mm^2^. Patient sex, systemic disease, and lifestyle habits, such as smoking and alcohol consumption, had no effect on ECL (Table 4).

**Table 4:** Recipient factors for graft endothelium attenuation.

		ECL rate of >30% at 1 month postoperative	Risk Ratio	*P* Value	ECD of <1000 cells/mm2 at 1 year postoperative	Risk Ratio	*P* Value	ECD of >2000 cells/mm2 at 1 year postoperative	Risk Ratio	*P* Value
**Gender**										
	Male	39/149 (26.2%)	0.6728	0.075	51/149 (34.23%)	1.542	0.947	38/149 (25.50%)	1.5107	0.123
	Female	30/77 (39.0%)			25/77 (32.47%)			13/77 (16.88%)		
**Age at surgery (year**)										
	>60	37 /100 (37.0%)	1.4567	0.039	39 /100 (39.0%)	1.3279	0.026	21/100 (21.0%)	0.8819	0.09
	≤60	32 /126 (25.4%)			37 /126 (29.4%)			30/126 (23.8%)		
**Systemic disease**										
	Yes	8/ 25 (32.0%)	1.0543	0.866	10/25 (40.0%)	1.2184	0.475	7/25 (28.0%)	1.2791	0.573
	No	61/ 201 (30.4%)			66/201 (32.8%)			44/201 (21.89%)		
**Smoke**										
	Yes	12 /52 (23.1%)	0.7042	0.186	19 /52 (36.5%)	1.1154	0.613	12/52 (23.1%)	1.029	0.92
	No	57 /174 (32.8%)			57 /174 (32.8%)			39/174 (22.4%)		
**Drink**										
	Yes	16/47 (34.04%)	1.1496	0.484	20 /47 (42.6%)	1.3603	0.148	9/47 (19.2%)	0.8163	0.53
	No	53/ 179 (29.61%)			56/179 (31.3%)			42/179 (23.5%)		
**Indications**										
	Infectious keratopathy	29/127 (22.8%)	0.5651	0.04	38/127 (29.9%)	0.7796	0.182	38/127 (30.0%)	2.4039	0.001
	Noninfectious keratopathy	40/99 (40.4%)			38/99 (38.4%)			13 /99 (13.1%)		
**Indications**										
	Bullous keratopathy	18/47 (38.3%)	1.2039	0.194	24/47 (51.1%)	1.7577	0.004	4/47 (8.5%)	0.3241	0.01
	Others	51/179 (28.5%)			52/179 (29.1%)			47/179 (26.3%)		
**Rejection**										
	Yes	NA	NA	NA	9 /15 (60.0%)	1.8898	0.025	0/15 (0%)	0	0.03
	No	NA			67/211 (31.8%)			51/211 (24.2%)		

**Abbreviations:** ECD = Endothelial cell density, ECL = Endothelial cell loss.

#### Surgical factors

The combined operations had a significant effect on the ECL in the early postoperative period. Patients who underwent combined ECCE or IOL had a significantly higher rate of ECL at postoperative 1 month than other patients, while the effect was no longer significant at postoperative 1 year, indicating that excessive operations caused some acute damage to the graft endothelium, but had less effect on chronic loss of corneal endothelial cells. However, patients without a combined operation had a higher probability of maintaining a high level of graft ECD. The implant diameter also affected postoperative ECL. In patients with larger graft diameters, the attenuation of ECD is slower, which may be due to the more corneal endothelium carried on large-diameter grafts (Table 5).

**Table 5:** Surgical factors for graft endothelium attenuation.

		ECL rate of >30% at 1 month postoperative	Risk Ratio	*P* Value	ECD of <1000 cells/mm2 at 1 year postoperative	Risk Ratio	*P* Value	ECD of >2000 cells/mm2 at 1 year postoperative	Risk Ratio	*P* Value
**Concurrent procedures**										
	Yes	20/41 (48.8%)	1.8414	0.005	20/41 (48.8%)	1.6115	0.016	3/41 (7.3%)	0.282	0.01
	No	49/185 (26.5%)			56/185 (30.3%)			48/185 (26.0%)		
**ECCE**										
	Yes	17/28 (60.7%)	2.3119	0.001	12/28 (42.9%)	1.3261	0.269	3/28 (10.7%)	0.4418	0.109
	No	52/198 (26.3%)			64/198 (32.3%)			48/198 (24.2%)		
**IOL implantation**										
	Yes	11/20 (55.0%)	1.9531	0.013	9/20 (45.0%)	1.2669	0.26	2/20 (10.0%)	0.4101	0.112
	No	58/206 (28.2%)			67/206 (35.5%)			49/206 (23.8%)		
**Graft diameter (mm)**										
	>8	9/55 (16.4%)	0.4662	0.011	16/55 (29.09%)	0.8290	0.022	18/55 (32.73%)	1.6959	0.006
	≤8	60/171 (35.1%)			60/171 (35.09%)			33/171 (19.30%)		

**Abbreviations:** ECD = Endothelial cell density, ECL = Endothelial cell loss.

## Discussion

The ultimate goal of PKP is to maintain graft transparency for as long as possible. Analysis of the causes of PKP failure showed that immunological allograft rejection was the main cause, with 28.2% of failures, followed by surface diseases (17.8%) and endothelial decompensation without rejection (17.3%) [5].

Persistent ECL is a problem in the field of corneal transplantation. Studies have shown that ECD also decreases yearly after PKP with a low risk of immune rejection, and the median decrease was 70%, 5 years after transplantation [6]. Moreover, risk factors for the decline in graft ECD have been explored, including glaucoma, anterior iris adhesions, small implants, and previous graft failure [7].

The first year after surgery represents early phase ECL, and the following years represent late-phase ECL [8]. The early postoperative attenuation rate determines the survival time of the corneal graft to some extent [8]. The ECL rate is highest in the first year after PKP at approximately 40%, decreases to 4.2% per year 5–10 years after surgery, then stabilizes after 10 years, decreasing by approximately 12% 10–20 years after surgery. This rate of decline is not affected by immune rejection, cause of corneal pathology, or donor age [3].

The donor cornea undergoes several stages of extraction, preservation, surgery, and adaptation to the postoperative environment. Each stage strikes the corneal endothelium to some degree, with surgical trauma being an important factor in the rapid loss of the corneal endothelium. Gediz et al. [9] reported that patients who underwent other intraoperative surgical procedures had lower postoperative graft transparency rates and that risky surgical procedures included anterior vitrectomy, goniosynechiolysis, and membranectomy.

Lass et al. [10] studied 567 patients undergoing PKP and found that younger donors seem to be associated with higher ECD in the first 5 years, especially from donors aged <40 years. However, recent studies have illustrated the absence of statistically significant differences in postoperative rates of ECL between corneas stored in tissue culture medium and organ-cultured corneas [11], although storage time in organ culture might affect the quality of grafts [12]. Our data show that DET affects the endothelial attenuation rate within 1 month after surgery, but has no significant effect on ECD in the long-term after surgery. In addition, younger donors and optisol preservation solutions had higher odds of maintaining a higher postoperative ECD.

Adaptation to the microenvironment of the anterior chamber after transplantation is the main cause of the chronic loss of graft endothelium within one year after surgery [13,14]. Yagi-Yaguchi et al. [15] discussed the effect of the level of preoperative inflammatory factors in the anterior chamber on ECD after PKP. It was found that patients with low ECD (ECD < 1200 cells/mm^2^) 6 months after PKP had high IL-6, IL-10, MCP-1, IFN-γ, and P-selectin expression, suggesting that inflammatory factors can affect the attenuation rate of corneal endothelial cells. Surprisingly, in our data, the rate of ECL was slower in patients with infectious keratopathy than in those with non-infectious keratopathy; whether the cause is related to cytokines in aqueous humor needs further discussion.

Large-diameter grafts carry more endothelial cells, and the ECL is slower than that of small-diameter grafts in the early postoperative period; however, large-diameter grafts have a higher risk of immune rejection and therefore do not have an advantage in maintaining a higher ECD in the long-term after surgery; thus, the setting of graft size should also be selected with respect to the actual situation of the patient [11]. Immune rejection is a fatal threat to corneal endothelium, and this study showed that the incidence of rejection within 1 year was 6.6% and that the ECD of grafts decreased to less than 1000 cells/mm^2^ in 60% of patients after rejection. It has also been reported that once rejection occurs, 30%–80% of endothelial cells are lost [16].

The present study indicates that postoperative ECL is faster in patients with corneal endothelial decompensation, which is consistent with the report by Chung et al. [17]. They also compared the changes in corneal endothelium after PKP in patients with corneal endothelial decompensation and keratoconus and found that the difference was not significant in the first 3 months postoperatively, while the ECL was significantly faster in patients with corneal endothelial decompensation after 3 months postoperatively. This may be related to the migration of the donor endothelium to the recipient’s peripheral cornea. Through scanning electron microscopy, Regis-Pacheco and Binder directly documented cell migration across the wound onto the host. The cells become larger after crossing the wound to cover the area where the endothelium is lacking [18].

Studies have shown that smoking and alcohol consumption also affect the corneal endothelium, with smokers having a significantly larger mean corneal endothelial cell size and significantly lower ECD than nonsmokers [19]. The number of hexagonal cells in the corneal endothelium was also significantly lower [20]. In contrast, corneal thickness and ECD were significantly lower in alcohol-dependent patients than in normal controls [21]. In the present study, the graft ECL rate was not significantly higher in smokers and alcohol-dependent patients in the early postoperative period, which may be related to the short exposure time.

Several studies have confirmed that diabetes has a detrimental effect on the number, morphology, and function of the corneal endothelium [22,23]. Therefore, it may seem that diabetes is a risk factor for ECL after PKP. However, the long-term effects of donors from diabetic patients on graft failure and ECD after PKP were investigated, and it was found that the 10-year graft failure rate and endothelial cell changes were not significantly different between diabetic and non-diabetic donors [24]. This study investigated the rate of ECL in patients with diabetes and found no significant differences when compared with normal subjects.

In conclusion, the rate of ECL after PKP is influenced by several factors, and *ex vivo* preservation and surgical stimulation cause rapid apoptosis and necrosis of a portion of endothelial cells, resulting in a dramatic decrease in ECD in the early postoperative period. Thereafter, the process of adaptation to the anterior chamber environment and migration of endothelial cells to the host can lead to further loss of graft endothelial cells. In contrast, the use of younger donors, minimizing unnecessary operation in the anterior chamber, and minimizing immune rejection may maintain a greater donor corneal endothelium in the long term.

## Supporting information

S1 Dataset Relevant data underlying the findings described in manuscript. (XLSX)
